# Plasticity of the Immune System in Children Following Treatment Interruption in HIV-1 Infection

**DOI:** 10.3389/fimmu.2021.643189

**Published:** 2021-07-29

**Authors:** Katrine Schou Sandgaard, Ben Margetts, Teresa Attenborough, Triantafylia Gkouleli, Stuart Adams, Mette Holm, Diana Gibb, Deena Gibbons, Carlo Giaquinto, Anita De Rossi, Alasdair Bamford, Paolo Palma, Benny Chain, Athina S. Gkazi, Nigel Klein

**Affiliations:** ^1^Great Ormond Street Institute of Child Health, University College London, London, United Kingdom; ^2^Department of Pediatrics and Adolescent Medicine, Aarhus University Hospital, Aarhus, Denmark; ^3^Molecular Haematology, Great Ormond Street Hospital for Children, London, United Kingdom; ^4^UCL Centre for Computation, Mathematics, and Physics in the Life Sciences and Experimental Biology (CoMPLEX), London, United Kingdom; ^5^Medical Research Council Clinical Trials Unit, London, United Kingdom; ^6^Peter Gorer Department of Immunobiology, Kings College London, London, United Kingdom; ^7^Department of Mother and Child Health, University of Padova, Padova, Italy; ^8^Section of Oncology and Immunology, Department of Surgery, Oncology and Gastroenterology, University of Padova, Padova, Italy; ^9^Immunology and Molecular Oncology Unit, Veneto Institute of Oncology IOV – IRCCS, Padova, Italy; ^10^Clinical and Research Unit of Clinical Immunology and Vaccinology, Academic Department of Pediatrics, Children Hospital Bambino Gesù - IRCCS, Rome, Italy; ^11^Division of Infection and Immunity, University College London, London, United Kingdom; ^12^Zayed Centre for Research into Rare Disease in Children, University College London, London, United Kingdom

**Keywords:** HIV-1, T cells, thymic output, antiretroviral treatment interruption, T cell receptor, immune repertoires, T cell receptor clonal expansions, high throughout sequencing

## Abstract

It is intriguing that, unlike adults with HIV-1, children with HIV-1 reach a greater CD4^+^ T cell recovery following planned treatment cessation. The reasons for the better outcomes in children remain unknown but may be related to increased thymic output and diversity of T cell receptor repertoires. HIV-1 infected children from the PENTA 11 trial tolerated planned treatment interruption without adverse long-term clinical, virological, or immunological consequences, once antiretroviral therapy was re-introduced. This contrasts to treatment interruption trials of HIV-1 infected adults, who had rapid changes in T cells and slow recovery when antiretroviral therapy was restarted. How children can develop such effective immune responses to planned treatment interruption may be critical for future studies. PENTA 11 was a randomized, phase II trial of planned treatment interruptions in HIV-1-infected children (ISRCTN 36694210). In this sub-study, eight patients in long-term follow-up were chosen with CD4^+^ count>500/ml, viral load <50c/ml at baseline: four patients on treatment interruption and four on continuous treatment. Together with measurements of thymic output, we used high-throughput next generation sequencing and bioinformatics to systematically organize memory CD8^+^ and naïve CD4^+^ T cell receptors according to diversity, clonal expansions, sequence sharing, antigen specificity, and T cell receptor similarities following treatment interruption compared to continuous treatment. We observed an increase in thymic output following treatment interruption compared to continuous treatment. This was accompanied by an increase in T cell receptor clonal expansions, increased T cell receptor sharing, and higher sequence similarities between patients, suggesting a more focused T cell receptor repertoire. The low numbers of patients included is a limitation and the data should be interpreted with caution. Nonetheless, the high levels of thymic output and the high diversity of the T cell receptor repertoire in children may be sufficient to reconstitute the T cell immune repertoire and reverse the impact of interruption of antiretroviral therapy. Importantly, the effective T cell receptor repertoires following treatment interruption may inform novel therapeutic strategies in children infected with HIV-1.

## Introduction

The interruption of antiretroviral therapy (ART) was explored as a way of boosting anti-HIV-1 immunity by increasing expression of HIV-1 antigens. However, in adults, ART interruption led to a rapid decline in the number of CD4^+^ T cells and increased mortality and morbidity (1). In contrast, in the PENTA 11 phase II randomized trial of planned treatment interruption (PTI) in children, ART interruption in children was better tolerated (2, 3). Although ART interruption was followed by a rapid reduction in circulating CD4^+^ T cells, CD4^+^ T cell numbers were maintained during the period of ART interruption at 48 weeks (4). Over two-thirds of children, despite the relatively high number with AIDS at start of ART, rapidly restored their CD4^+^ count following ART re-introduction with limited adverse clinical impact (2, 3). When all children in the PTI arm had been back on ART for at least 5 years, there was little evidence that children had suffered any long-term clinical, virological, or immunological consequences (5). The reason for better outcomes in children remain unknown, but may be related to differences in the homeostasis of their T cell repertoire. In adults, the maintenance of T cells in the periphery occurs predominantly by redistribution of cells from tissues to blood, with a small and very slow recovery in the naive repertoire, largely due to proliferation of existing T cell clones with limited thymic production (6). In contrast, young children have a very high thymic output of naïve T cells, peaking at 1–2 years, then gradually declining to much lower levels by early adulthood, resulting in dramatic reduction of T cell receptor (TCR) diversity with age (7–11). We undertook this study to identify the processes which drive T cell development following ART interruption and reintroduction, to inform future HIV-1 treatment strategies in children. The low sample size of this study is a limitation that precludes definitive statistically analysis, so the results and conclusions will require validation in a larger cohort.

## Materials and Methods

### Participants and Samples

PENTA 11 was an open, multicenter, randomized, phase II trial (ISRCTN 36694210) in HIV-1-infected children aged 2 to 15 years on any ART regimen that included at least three drugs for at least 24 weeks before the beginning of the study (4). Eligibility criteria were (1) two most recent plasma HIV-1 RNA <50 copies/ml (screening and pre-screening), (2) CD4% ≥30% (ages 2–6 years) or ≥25% and CD4 count ≥500 cells/mm^3^ (7–15 years). For our sub-study, eligibility criteria were the inclusion of longitudinal UK samples, viable cells with at least 5 million PBMCs and CD4^+^ count>500/ml and viral load <50c/ml. We found 12 patients in total; however, four were excluded due to low amounts of viable PBMCs in the thawed samples. Eight patient samples met the eligibility criteria: four patients on PTI and four on continuous treatment (CT) (**Table 1**). In the PTI group, cells were available from before PTI (week 0, when ART was stopped), during PTI (week 12), at the end of PTI before ART re-initiation (three patients restarted ART at 48 weeks and one patient (patient 1) restarted ART at 24 weeks), and after PTI (long term follow-up – 3 years after end of study). ART was restarted if confirmed CD4% was less than 20% or more than 48 weeks had been spent off ART. The one patient that restarted ART at week 24 met the low CD4% outcome at week 24. In the CT group, samples were analyzed from the corresponding weeks 0, 48, and 150. The majority of the patients had a record of EBV and CMV exposures.

**Table 1:** Patient Characteristics.

	Patients with PTI*	Patients with CT**
No. of participants	4	4
Age in years, median (IQR***)	9·5 (8·97-10)	8 (7·5-9·8)
CD4^+^ count/ul at baseline, median (IQR)	965 (870-1085)	1365 (1197·5-1471)
Viral load/ml at baseline, median (IQR)	50 (50-50)	50 (50-52·5)

*PTI, Planned treatment interruption.

**CT, Continuous treatment.

***IQR, Interquartile range.

The protocol for this study was approved by the ethics committee for each participating center, and informed written consent was obtained from all participants and their parents or guardians (Great Ormond Street Hospital, St Mary’s Hospital and Chelsea and Westminster Hospital), approval from Trent MREC.

### Fluorescence-Activated Cell Sorting (FACS) of T Cell Sub Populations

**Figure 1:**
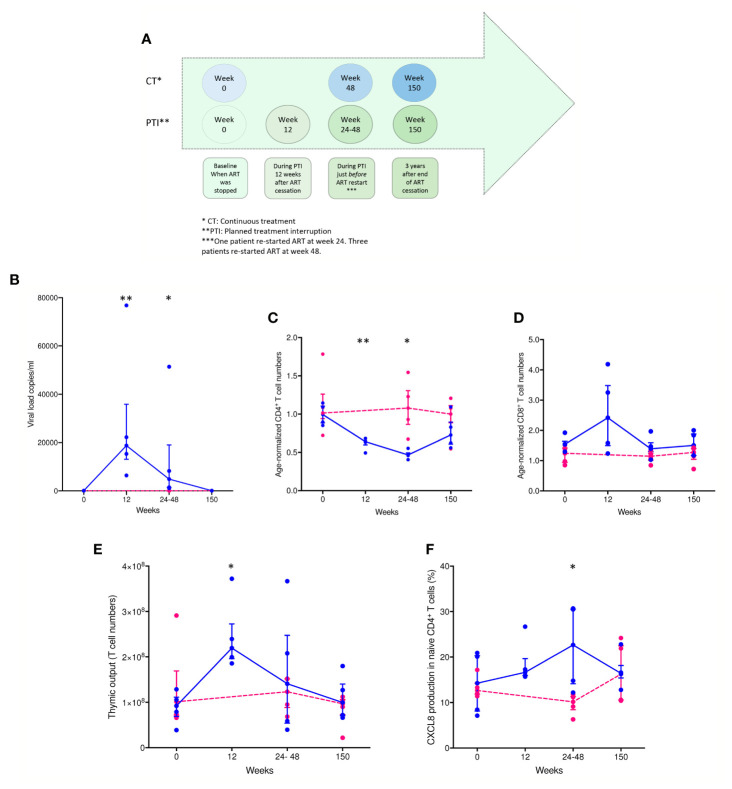
Changes in viral load, age-normalized CD4^+^ and CD8^+^ T cell numbers, thymic output and CXCL8 T cell production over time in children with HIV. **(A)** Timeline of collected patient samples. **(B)** Viral load (copies per ml). **(C)** Age-normalized CD4^+^ T cell counts. **(D)** Age-normalized CD8^+^ T cell counts. **(E)** Thymic output (number of recent thymic emigrants measured by the mathematical model). **(F)** CXCL8-production in naïve CD4^+^, CD45RA^+^, CD31^+^ T cells (%). CT children [pink individual measures and dotted lines connecting median(IQR)] and PTI children [blue individual measures and solid lines connecting median(IQR)]. *P ≤ 0.05, **P ≤ 0.005.

Total PBMCs were thawed and washed in RPMI 1640 medium (Invitrogen) containing 10% fetal calf serum (StemCell Technologies), 2 mM L-Glutamine (Sigma), 100 U penicillin, and 100 µg/ml streptomycin (Invitrogen) (complete medium, CM). Thawed PBMCs were FACS sorted to isolate naïve CD4^+^, CD45RA^+^, CD45RO^-^ T cells and memory CD8^+^, CD45RO^+^, CD45RA^-^ T cell subsets for downstream high throughput sequencing with T cell specific primers. Antibodies used were CD4-APC (BD-Biosciences), CD45RA-Vio700 (BD-Biosciences), CD8-APC-Cy7 (BD-Biosciences), and CD45RO-PE (BD-Biosciences) with a live/dead stain (AQUA, Invitrogen). PBMCs were fixed using Cell Fix (BD-Biosciences). The gating strategy is shown in **Figure 1S** (in Supplementary). Samples were analyzed on a FACSAria III cell sorter using FACSDiva software v.8.0. The purity of each carefully separated and compensated T cell group: the naïve CD4^+^, CD45RA^+^, CD45RO^-^ T cells and memory CD8^+^, CD45RO^+^, CD45RA^-^ T cell subsets, were 95-100%.

### Cell Stimulation for CXCL8 Detection

A potential new marker for thymic output is interleukin-8 or chemokine (C-X-C motif) ligand 8 (CXCL8), which has been shown to demonstrate an important T cell effector function in human infants (12, 13). Pediatric naïve CD4^+^ T cells have an enhanced capacity to produce CXCL8, with highest levels in infants that decrease rapidly with age corresponding to the decline in thymic output with age (12). In addition, CXCL8 producing T cells contain more TRECs than CXCL8 non-producing cells. PBMCs were stimulated with phorbol myristate 13-acetate (PMA) (Sigma 10ng/ml) and ionomycin (1 µg/ml Sigma) in the presence of brefeldin A (BFA) (20 µg/ml Sigma) in CM for 3.5 hours at 37°C with 5% CO_2_ before staining with CXCL8.

### Flow Cytometry

PMA/ionomycin-stimulated PBMCs were stained using fixable AQUA live/dead stain (Invitrogen) and the following antibodies: CD3-BUV395 (Invitrogen), CD4-BV605 (BD Biosciences), CD45RA-PerCPVio700 (BD Biosciences), CD31-BV421 (BD Biosciences), CD45RO-PE (BD Biosciences), and CD8-APC-Cy7 (BD Biosciences) in FACS buffer (phosphate-buffered saline containing 0.2% bovine serum albumin and 0.02% sodium azide). PBMCs were fixed and permeabilized (Foxp3, eBioscience). The PBMCs were intracellular stained with Ki67-AF647 (BD Pharmigen, measured in un-stimulated PBMCs) and IL-8-AF488 (CXCL8, BD Biosciences, measured in stimulated PBMCs) and incubated in the dark on ice for 1 hour. Samples were analyzed on an LSRII (Becton Dickinson) using FACSDiva software v.8.0. Subsequent data analysis was performed using FlowJo software 10.4.

### Real-Time PCR for TRECs

Real-time quantitative polymerase chain reaction (qPCR) was carried out for T cell receptor excision circles (TRECs) and a human control gene (*TRAC*) on a TaqMan 7500 Fast system (Applied Biosystems) using 12.5 µl of TaqMan Universal Master Mix and 5 µl of DNA with primers and probes as previously described (14). Standard qPCR cycling conditions were used. A standard curve was generated for TRECs and *TRAC* using serial dilutions of the TREC/KREC/*TRAC* plasmid. The number of TRECs per cell was calculated using the copy number ratio of TRECs: *TRAC* and multiplying by 2 to account for the presence of two copies of the *TRAC* gene in a diploid cell.

### Modeling Thymic Output

A model-based method for estimating thymic output and its validation using thymus size and cellularity from 0 to 20 years of age has previously been described in detail (7). Briefly, an explicit expression for thymic export in terms of total naïve CD4^+^ cell numbers, naïve CD4^+^ T cell TREC content, and naïve CD4^+^ T Ki67 expression is given as:

Thymic export (cells/day)=θ(t) 

$$\;=\left(\frac{y(t)\tau (t)}{\Delta }+\frac{d\tau (t)}{dt}\right)\frac{N(t)}{c-\tau (t)}$$

where *c* is a constant representing the average TREC content of thymic emigrants entering the peripheral naïve T cell population, *y*(*t*) is the flow cytometry-estimated Ki67 fraction of naïve CD4^+^ T cells, Δ is the duration of Ki67 expression, τ is the qPCR-estimated TREC concentration in the sorted naïve CD4^+^ T-cell population, and *N*(*t*) is the total size of the naïve CD4^+^ T-cell pool calculated from the clinical trial unit’s lymphocyte counts from fresh PBMC samples. Estimated total body CD4^+^, CD45RA^+^ T cell numbers *N(t)* were calculated as previously described, referring to the linear relationship between blood volume and body weight (15, 16). Parameter values *c* and Δ were obtained as described previously (7).

### Genomic DNA and RNA Isolation From FACS-Sorted Fixed Cells

We developed a modified protocol for isolation of DNA and RNA from fixed cells as previously published (17). Genomic DNA was extracted from low numbers (50,000–200,000) of naïve CD4^+^ T cells using the DNA Microextraction Kit (QIAGEN). Briefly, cells were re-suspended in 100 µl buffer ATL (lysis buffer), 10 µl proteinase K and incubated at 56°C for 1 hour following 90°C for 1 hour to reverse the partial formaldehyde modification of the nucleic acids. Genomic RNA from sorted naïve CD4^+^ and memory CD8^+^ T cells was extracted in two steps. In the first step the formalin-fixed, paraffin-embedded tissue RNeasy kit (QIAGEN) was used. The cells were re-suspended in 150 µl Proteinase K digestion buffer, 10 µl proteinase K and incubated at 56°C for 15 minutes, following incubation at 80°C for 15 minutes. Next, the RNeasy Micro kit (QIAGEN) was used. RNA quantity and quality were verified on a Nanodrop and Qubit fluorometer (Thermo Fischer Scientific).

### TCR Repertoire Sequencing

TCR repertoire libraries were prepared using a RNA-based 3’ RACE protocol incorporating unique molecular identifiers for quantitative TCR sequencing thus corrected for PCR amplification bias. The unique molecular identifier (UMI)-based RACE protocol is now considered gold standard. The method provides a template switch effect allowing continuous replication of the entire oligonucleotide. It captures all TCR variants present in the sample and only one primer set is required per reaction avoiding use of multiple primer sets and thus the association of the linked amplification bias. Full details of the method have recently been published (18–20). Up to 12 final amplicon products were pooled together and loaded, at 12pM concentration, on an Illumina MiSeq, using a version 2 chemistry 2x250PE kit.

### Data Analysis

The FASTQ files produced on the MiSeq were processed using the Decombinator package (21, 22). Decombinator, which incorporates barcode dependent correction for sequencing error and PCR bias, annotates each TCR sequence according to V gene and J gene usage, and the highly variable complementary-determining region 3 (CDR3) sequence. The output from Decombinator is then grouped according to UMIs which is incorporated into each cDNA molecule by the ligation step. Within multiple identical TCR sequences in the same UMI group of equal abundance one is chosen arbitrarily and the rest discarded. The barcode information is essential since the probability of two identical TCRs having the same 12-mer barcode is very low. Identical TCR sequences in the same UMI group are discarded since they were most likely derived from the same TCR cDNA molecule by PCR/sequence error. The number of different UMIs paired with a single identical TCR sequence provides the frequency of the sequence in the sample.

The distribution of TCR abundances in each repertoire was summarized using the Gini coefficient, a measure of distribution inequality. The scale ranges between 0 and 1, where 0 = completely equal (x clones, all with identical frequencies) and 1 = completely unequal (i.e., tending toward sample oligoclonality). Because the Gini coefficient is affected by total population size, all repertoires were subsampled to the same number of reads. Minimum subsampling depth for each sample was algorithmically determined to preserve sample distribution in each individual and then transcript abundance was compared in single individuals or across individuals by subsampling all transcripts down to the same number preserving sample distribution as previously published (20). Gini indices were computed using the “ineq” package (version 0.2.13) in the R environment (version 3.3.2).

For clustering TCRs that are predicted to bind the same MHC-restricted peptide antigen, we utilized the “Grouping Lymphocyte Interactions by Paratope Hotspots” (GLIPH) algorithm (23). The algorithm returned lists of significant, locally enriched motifs of continuous amino acids and combined enriched motifs, CDR3 length, V gene, and J gene usage with clonal CDR3 expansions, creating sets of related TCRs likely to recognize the same antigen. TCR CDR3 sequence similarities were also computed using the Hamming distance and a shared triplet metric as previously reported (24).

The R package ggplot2 (version 2.2.1) and Prism (version 8) were used for all data visualizations. All manual data analyses were executed using custom scripts written in R (version 3.3.2) and Python (version 3.7.3). The Decombinator package v3.1 was executed using Python (version 2.7.3). All software is freely available at https://github.com/innate2adaptive/Decombinator.

The statistical test used for comparisons between PTI and CT groups was an unpaired *t*-test using Welch’s correction. The statistical test used for comparisons between PTI time points was a paired *t*-test. The low numbers of patients included is a limitation, which precludes a clear and statistically significant conclusion.

## Results

### Thymic Output Increases Following ART Interruption

Samples were obtained as shown in **Figure 1A**. ART interruption led to a significant rise (P < 0.0014) in viral load from week 0 to week 12 in the PTI group. The increase in viral load was significant in PTI compared to CT in week 24-48 (P < 0.0140), which was reversed after antiretroviral treatment was resumed (**Figure 1B**). In parallel, ART interruption led to a rapid drop in CD4^+^ T cells from week 0 to week 12 in the PTI group (P < 0.0023), and in comparison to CT week 24-48 (P < 0.0286). The drop in CD4^+^ T cells had not completely reversed at 3 years after re-initiation of ART (**Figure 1C**). Age normalized T cell numbers were calculated from published age-matched lymphocyte population reference values (25). ART interruption was also accompanied by a transient rise in CD8^+^ T cells (not significant, but an increasing trend: P = 0.0571, when PTI is compared to CT week 24-48), in thymic output from baseline to week 12 in PTI (P < 0.0341), and in proliferation (not significant) (**Figures 1D–F** and **Table 1S**) (7, 15, 16). Further, ART interruption led to a significant increase in CXCL8 in PTI compared to CT week 24-48 (P < 0.0403) (**Figure 1F**). In contrast, individuals with continuous ART did not show changes in any of the measured parameters over the same period.

### ART Interruption Drives Increased Clonal Expansion in the Naïve T Cell Population

**Figure 2:**
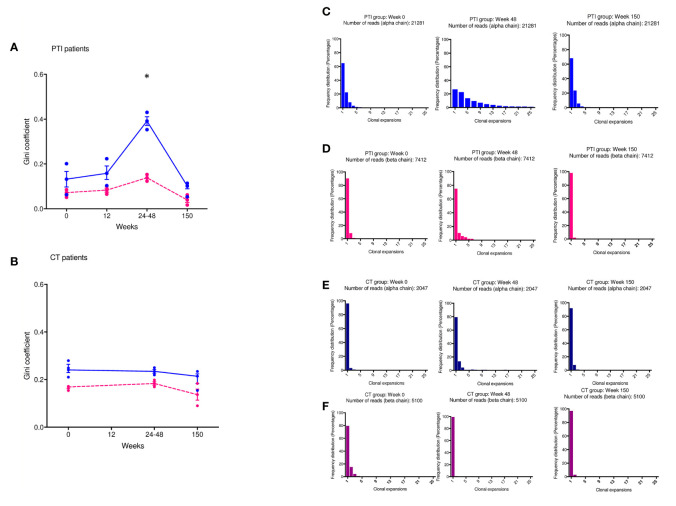
Changes in TCR abundance distribution in naïve CD4^+^ T cells during ART treatment interruption. **(A, B)** The degree of clonal expansion in the TCR repertoire measured using the Gini coefficient in TCRα (blue) and TCRβ sequences (pink) in the PTI **(A)** and CT **(B)** group. **(C–F)** TCR sequence abundance distribution in representative individuals. **(C, E)** TCRα; **(D, F)** TCRβ. **(C, D)** PTI group; **(E, F)** CT group. TCRβ [pink individual Gini coefficients and dotted lines connecting median(IQR)] and TCRα [blue individual Gini coefficients and solid lines connecting median(IQR)]. *P ≤ 0.05.

We next examined the impact of treatment interruption on the CD4^+^ naïve TCR repertoire clonal abundance distribution. The Gini coefficient in the naïve T cell population increased significantly by the end of the interruption period compared to CT in week 24-48 (P < 0.0173), suggesting clonal expansion, but had returned to baseline by 3 years (**Figure 2A**). The Gini index showed no changes in the children maintained continuously on ART (**Figure 2B**). The Gini index of the TCRα chain was consistently greater than the β chain, reflecting the lower diversity of TCRα because of the absence of a D region. The number of expanded TCRs in the naïve repertoires increased following ART interruption, reflected in the abundance distribution (number of times the same TCR sequences are observed) for individual repertories (**Figures 2C–F**). In two individuals, PTI was followed by a transient increase in the number of more abundant TCR sequences, but the distribution had returned to the pre-PTI profile by 3 years post-interruption (**Figures 2C, D**). No changes in TCR abundance distribution were observed in the children maintained continuously on ART (**Figures 2E, F**). In contrast to the CD4^+^ naïve population, no change in Gini index or TCR abundance distribution was observed in the CD8^+^ memory population. As expected, the memory CD8^+^ population had a greater proportion of abundant TCRs at all time points (**Figure 2S**) (18).

### ART Interruption Is Associated With a New CDR3 Repertoire Composition

**Figure 3:**
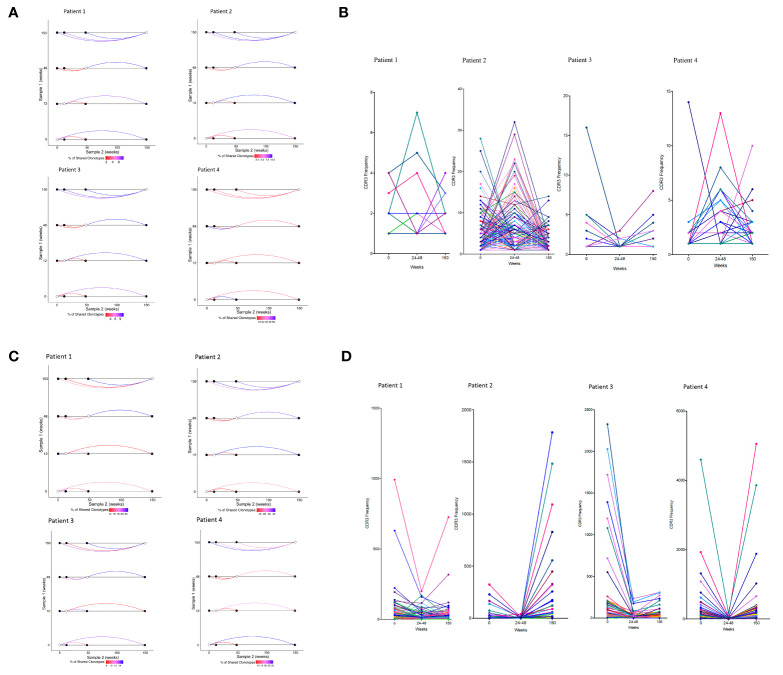
Repertoire dynamics and sequence sharing associated with ART interruption. **(A)** Shared CDR3s between two time points in individual patients following PTI in naïve CD4^+^ TCRs and **(C)** memory CD8^+^ CDR3s. Shown is the proportion (%) of shared TCRα CDR3s by the color of the connecting line between two time points. The lines are colored red for low sharing and blue for higher sharing. **(B)** The CDR3 frequency dynamics in individual patients of shared CDR3s in the naïve CD4^+^ TCR repertories following PTI. **(D)** The CDR3 frequency dynamics in individual patients of shared CDR3s in the memory CD8^+^ TCR repertories following PTI.

Approximately 2–10% of TCRα sequences and 2–5% of TCRβ sequences in the naïve CD4^+^ repertoire was shared between at least two time points in three of the PTI patients (**Figure 3A** and **Figure 3SA**). However, the degree of CDR3 sharing between time points fell dramatically following the start of PTI compared to baseline (seen by the red lines), suggesting PTI induced dynamic changes in repertoire composition. In contrast, many sequences were shared between the samples at the end of PTI and when taken 3 years later, attesting to the stability of the new clonally expanded CDR3s and established repertoire diversity. The new CDR3 composition following PTI resembling the long term CDR3 composition could be a result of ART interruption. The dynamics of individual TCRs that were shared between at least three time-points is shown in **Figure 3B**. In three patients, the shared TCRs increased in abundance following PTI, suggesting clonal expansions were starting to occur in the naïve T cell population. The CD8^+^ memory compartment had 10–40% sharing between time points (**Figure 3C** and **Figure 2SB**), greater than in the CD4^+^ naïve population. However, as in the naïve repertoires, the degree of sharing largely decreased following the start of PTI, and the established TCR repertoire by the end of PTI was maintained 3 years after end of study. In contrast to the naïve CD4^+^ compartment, the abundance of many of the shared CD8^+^ memory CDR3s decreased in frequency following the start of PTI (**Figure 3D**).

### The Dynamics of Shared (Public) TCRs Following ART Interruption

**Figure 4:**
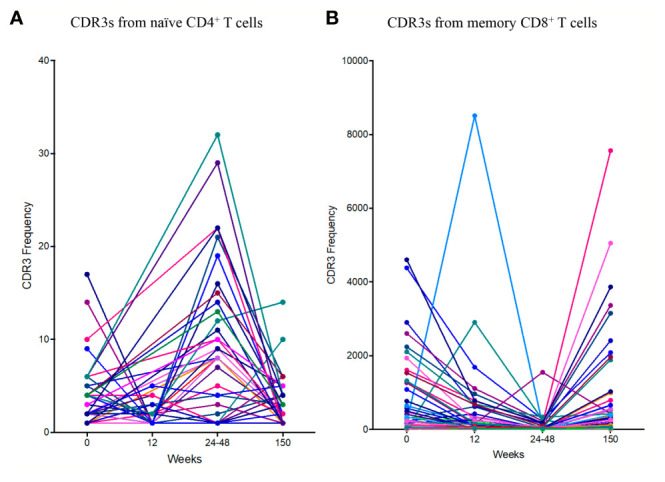
The dynamics of the public TCR repertoire during PTI. The y axis shows the abundance of TCRs shared by all four PTI individuals at one or more time points. **(A)** Naïve CD4^+^ TCR repertoires and **(B)** Memory CD8^+^ TCR repertoires. Annotated CDR3s from the VDJ database are listed with corresponding amino acid sequences, epitopes, TCR population and chain (**Table 2**).

We identified a significant number of TCRs that were found in all four PTI patients at least at one time point (public TCRs). We plotted the dynamics of these TCRs across time (**Figures 4A, B**). In the naïve CD4^+^ TCR repertoire, a transient increase in the abundance of the public TCRs was found following PTI, most evident at 24–48 weeks after ART cessation (**Figure 4A**). The expanded TCRs shared by all PTI patients included some sequences as being part of anti-CMV or anti-EBV TCR receptors annotated from the VDJ database (**Table 2**). HIV-1 specific TCR sequences annotated from the VDJ database were found in each individual, however the TCRs were not shared by all 4, hence not shown in **Table 2**. In contrast, shared memory CD8^+^ TCRs were found to be less abundant following PTI (**Figure 4B**). The shared TCRs in the memory compartment included more sequences annotated as CMV specific compared to the naïve CD4^+^ TCRs, perhaps reflecting the high prevalence of CMV exposure. The decrease in abundance of public TCRs is also consistent with widespread replacement of the non-HIV-1 expanded memory repertoire by HIV-1-specific T cells responding to the increased levels of HIV-1 antigens during PTI. Only one patient from the CT group had sufficient samples to allow a direct comparison to the PTI group. This CT patient did show fewer shared sequences with low frequencies than observed in the PTI group.

**Table 2:** VDJ database annotated CDR3s from memory CD8^+^ T cells shared between all four PTI patients

CDR3	Epitope species	T cell type	Chain
CAVLDSNYQLIW	*Homo sapiens*	CD8^+^, CD45RO^+^	α
CAVMDSNYQLIW	CMV/*Homo sapiens*	CD8^+^, CD45RO^+^	α
CAVLDSNYQLIW	*Homo sapiens*	CD8^+^, CD45RO^+^	α
CAVRDRDYKLSF	CMV	CD8^+^, CD45RO^+^	α
CAVRDSNYQLIW	CMV	CD8^+^, CD45RO^+^	α
CAPMDSNYQLIW	Influenza A	CD8^+^, CD45RO^+^	α
CAVYQAGTALIF	Influenza A	CD8^+^, CD45RO^+^	α
CAGMDSNYQLIW	*Homo sapiens*	CD8^+^, CD45RO^+^	α
CAVSDSNYQLIW	Influenza A	CD8^+^, CD45RO^+^	α
CAVTDSNYQLIW	*Homo sapiens*	CD8^+^, CD45RO^+^	α
CAVRDGDYKLSF	CMV	CD8^+^, CD45RO^+^	α
CAATDSNYQLIW	CMV	CD8^+^, CD45RO^+^	α
CASMDSNYQLIW	Yellow Fever Virus	CD8^+^, CD45RO^+^	α
CAVMDSNYQLIW	CMV/*Homo sapiens*	CD8^+^, CD45RO^+^	α
CAFMDSNYQLIW	Influenza A	CD8^+^, CD45RO^+^	α
CATMDSNYQLIW	CMV	CD8^+^, CD45RO^+^	α

### ART Interruption Is Associated With the Emergence of TCR Clusters

**Figure 5:**
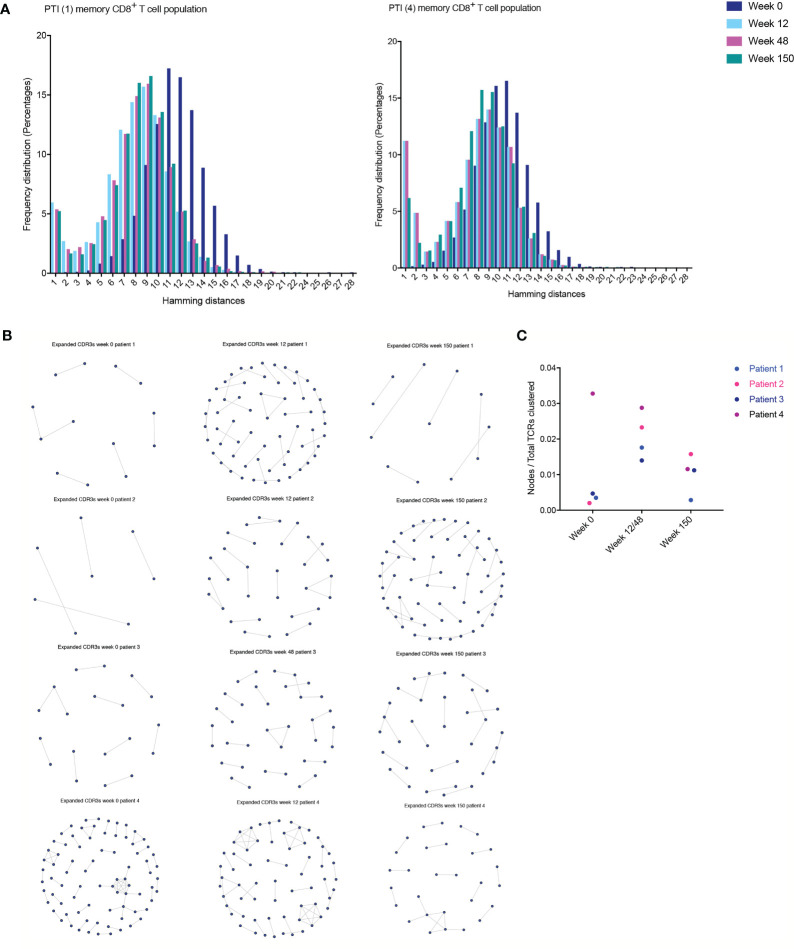
T cell clustering following ART interruption. TCR CDR3 sequence similarities were computed using the Hamming distance to tests two pairwise CDR3s of equal length at a time creating a CDR3 similarity network. A Hamming distance of 1 reflects a single AA change between two neighboring CDR3s. **(A)** The distribution of pairwise Hamming distances between CDR3s in the memory CD8^+^ T cell repertoires. **(B)** TCR CDR3 sequence similarities were also computed using a shared triplet metric of closely related CDR3s forming network clusters in the memory CD8^+^ T cell population. **(C)** The number of nodes as a proportion of the number of total TCRs being clustered for each sample following PTI.

Antigen specific responses are often associated with the expansion of clusters of closely related TCRs (23, 26). Closely related TCRs have similar CDR3 regions with the same continuous amino acids (AA) in their sequences called motifs. To examine sequence similarity, we first computed the Hamming distance metric showing the number of AA differences between CDR3 sequences. The model tests two pairwise CDR3s of equal length at a time, creating a CDR3 similarity network in our cohort. A Hamming distance of 1 reflects a single AA change between two neighboring CDR3s. While concentrating our investigation on antigen-exposed TCRs in the memory CD8^+^ T cell population, we observed an increase of more “similar” CDR3s (low Hamming distance) in two of the patients (1 and 4) following PTI. (**Figure 5A**). The remaining two patients remained consistent throughout all sampling times.

TCRs were then clustered according to their CDR3 sequence similarity and motif conservation using the “Grouping Lymphocyte Interactions by Paratope Hotspots” (GLIPH) algorithm (23). GLIPH predicts which TCRs are able to bind the same MHC-restricted peptide antigens. First, the algorithm returns lists of significant, locally enriched motifs of AA found within thousands of similar CDR3s. We found an increased number of enriched AA motifs in the CDR3 sequences following PTI in three of the patients (**Table 3**). The GLIPH algorithm then estimates the number of similar “antigen specificity groups” within the memory TCR repertoire (**Table 3**). Overall, we observed a decreased number of specificity groups following PTI in all four patients, reflecting a decreased TCR repertoire diversity returning to baseline levels in the long term.

**Table 3:** TCR clustering characteristics in the memory CD8^+^ T cell population.

Numbers of significantly enriched local motifs	Patient	Week 0	Week 12	Week 24-48	Week 150
	1	183	192	198	196
	2*	95	170	84	84
	3	138	125	114	138
	4	201	357	250	204
**Numbers of significant antigen specificity groups**					
	1	9,943,570	8,576,011	9,393,945	12,184,516
	2	952,891	1,483,503	56,616	105,761
	3	2,750,685	951,511	1,396,956	3,952,266
	4	6,703,291	8,576,011	5,666,661	9,881,235

*2 had lower number of reads compared to the other samples.

Finally, we looked for the presence of clusters of closely related CDR3s using a shared triplet metric of similarity (24). Again, the algorithm returns clusters of significantly enriched motifs of three AA. Using this algorithm, the clusters of related TCRs are represented as nodes in a network, and we observed an increase in clusters following PTI in patient 1,2 and 3 (**Figure 5B**). In Patient 4, we observed a higher number of clusters even before PTI, which is likely to reflect previous antigen exposure. We measured the number of TCRs in a cluster (the number of nodes in **Figure 5B**), as a proportion of the number of total TCRs being clustered for each sample. The metric is seen in **Figure 5C**; the increase following PTI is not significant (Two-way ANOVA **Table 2S**). If we exclude patient 4 on the grounds that it is an outlier, the increase following PTI was significant in patient 1,2 and 3 (P < 0.035).

## Discussion

In contrast to adults, children with HIV-1 have enhanced capacity to recover their CD4^+^ T cells following treatment interruption (1, 2). This present study is the first to report on the immunological events that occur during antiretroviral treatment interruption and overall demonstrates that children were able to restore immune repertoires following ART re-introduction.

The observed T cell numbers and thymic output measurements were broadly in agreement with previous studies (7, 11, 13, 27). In recent analysis, the majority of the patients, as we also observed, increase early in CD8^+^ counts and return to pre-PTI levels after ART re-initiation (5). However, there was a proportion of patients with lower CD4^+^ count who experienced a slower CD8^+^ recovery once ART was restarted. In our sub-study, ART interruption resulted in a rapid increase in viral load and changes in thymic output, production of CXCL8, and changes in the TCR repertoire. It is well known that thymic output improves and continues to reconstitute with time on ART in children, suggesting that ART can either reverse or modulate HIV-1 induced injury to the thymic microenvironment (11). The capacity to increase thymic activity in children may be critical in the homeostatic replenishment of T cell development and function, and may explain why viral loads decreased, even before ART was re-started. The return of thymic output, CXCL8 production and TCR repertoires to pre- ART interruption levels is consistent with high capacity of children to recover immune function (3, 28). If the PTI period was further prolonged, the impact on thymic output could be less effective, largely as a consequence of HIV-1 disease progression.

Following PTI, we observed a transient increase in the Gini coefficient of the naïve CD4^+^ TCR repertoire, transient expansions of a set of naïve CD4^+^ cells, and perturbation of the repertoire. Clonal expansions in the naïve repertoire may reflect homeostatic expansion (increased Ki67) of the naïve T cell population driven by the rapid fall in CD4^+^ T cells and complementing increased thymic output (29). Interestingly, these effects of treatment interruption are all transient, and post-interruption naïve CD4^+^ TCR repertoires return to baseline levels over the next 3 years. Cross-contamination between naïve and memory T cell subsets were carefully considered by assuring purity of separated and precisely compensated naïve T cell subsets (activation marker CD45RO^-^) and memory T cell subsets (CD45RO^+^) being 95-100%. Further, it is highly unlikely that cross-contamination would appear only in the naïve PTI samples during NGS library preparation since these were not prepared together with the memory TCRs. While experimental contamination of CD45RA^+^ cells with CD45RO^+^ cells is unlikely to explain the repertoire changes we observed, we cannot exclude the possibility that there is some contribution of minor non-naïve CD45RA^+^ T cells, such as CD45RA^+^ EMRA (terminally differentiated effector memory cells re-expressing CD45RA) or stem memory (CD95^+^, CD45RA^+^) populations (30). Further more extensive phenotyping will be necessary to fully characterize the contribution of these populations.

PTI also induced changes in the memory TCR repertoire. The diminished number of memory TCRs following PTI could reflect the cells migrating back to other tissues such as the intestinal tract and lymph nodes. Interestingly, the TCR repertoire during PTI was more focused, and we observed the emergence of clusters of highly structurally related TCRs, indicating an antigen-specific response persisting over time (23, 26, 27, 31–33). As noted in **Table 3**, using the GLIPH approach shows a range of connected TCRs and plausible related motifs for potential targets such as HIV-1, CMV, and EBV (56,616–12,184,516). It is tempting to suggest that the TCR expansions, and the TCR clusters both reflect HIV-1-specific CD8^+^ response driven by the increased HIV-1 replication and consequent expression of HIV-1 antigens when ART was removed (31, 32). However, in relation to antigen specificity using the VDJdb, one single chain match does not conclude receptor specificity. Functional studies of T cell reactivity will be required to confirm the antigen-specificity of the T cells responding to ART interruption, although these are challenging to perform in blood samples taken from children.

The low numbers of patients included in this study is a limitation which precludes a robust and statistically significant conclusion. Statistical analysis comparing low numbers of samples should be interpreted with caution. Flow cytometry analysis and particularly the TCR repertoire sequencing analysis require a large amount of good quality RNA, and large numbers of T cells from big volumes of blood, all of which are difficult to obtain in a pediatric sample setting. Furthermore, longitudinal samples from children with HIV-1 during planned ART interruption are very rare and we therefore undertook this study, despite knowing the statistical and practical challenges. However, the longitudinal nature of this study with samples during PTI and long term follow-up were essential for the study of the immunological changes resulting from treatment interruption. Comparing the PTI samples with the CT samples increased the likelihood of the observed immunological changes being true changes in response to PTI. Whilst the few samples do not represent the entire PTI population, it was a unique chance to understand the T cell based immunological mechanisms behind the pediatric immune response to HIV-1. Recent work supports our findings, reporting that even after 5 years, children tolerated PTI with few long-term clinical, virological or immunological consequences (5). The present study provides insight to new mechanisms as to why children reconstitute their T cells effectively and provides opportunities for future care of children with HIV-1 infection.

In conclusion, our study documents profound changes in both the naïve and memory T cell compartment consequent to treatment interruption. These changes likely reflect both a homeostatic mechanism to preserve the naïve repertoire in the face of HIV-1-dependent T cell destruction and a strong antigen driven response to either HIV-1 itself or to increased exposure to other microbial antigens. Despite these major changes, the repertoire of the HIV-1-infected children proved remarkably robust to the effects of PTI, and almost all parameters we examined returned to pre-interruption levels 3 years after ART was reinstated. The increased plasticity of the immune system of children may afford them a better opportunity to deal with the immunological stress imposed by ART interruption, and cognizance of this may provide enhanced potential for novel treatment strategies in the future.

## Data Availability Statement

The datasets presented in this study can be found in online repositories. The names of the repository/repositories and accession number(s) can be found below: https://www.ncbi.nlm.nih.gov/, SRP199361.

## Ethics Statement

The studies involving human participants were reviewed and approved by the ethics committee for each participating center, and informed written consent was obtained from all participants and their parents or guardians (Great Ormond Street Hospital, St Mary’s Hospital and Chelsea and Westminster Hospital), approval from Trent MREC. Written informed consent to participate in this study was provided by the participants’ legal guardian/next of kin.

## Author Contributions 

KS performed the Flow cytometry and NGS experiments, applied the mathematical and bioinformatic analysis to the data, created the data presentation/visualization, and wrote the original draft. NK designed the study aims and acquired the financial support of the study. NK and AG oversaw and had leadership responsibility for the research planning and execution and contributed to drafting the manuscript. AG designed and supervised the NGS methodology. BC developed the NGS methodology. BC, BM, and TA supervised the bioinformatics. TG contributed to performing NGS experiments. SA designed and performed the TRECs analysis. DeG designed and supervised the CXCL8 methodology. BC, BM, TA, DG, and TG contributed to drafting the manuscript. MH, CG, AR, AB, and PP contributed to the conception and design of the study and writing the manuscript draft. All authors contributed to the article and approved the submitted version.

## Funding

This work was supported by Reuben Centre for Virology and Metagenomics, Action Medical Research, PENTA Foundation, ViiV, UK Medical Research Council, United Kingdom, the Health Research Foundation of Central Denmark Region, Aarhus University Research Foundation, and the Lundbeck Foundation, Denmark. The funding source had no involvement in the study design, data collection, analysis, interpretation or drafting the paper. The corresponding author confirms that she had full access to all the data in the study and had final responsibility for the decision to submit for publication.

## Conflict of Interest

The authors declare that the research was conducted in the absence of any commercial or financial relationships that could be construed as a potential conflict of interest.

## Publisher’s Note

All claims expressed in this article are solely those of the authors and do not necessarily represent those of their affiliated organizations, or those of the publisher, the editors and the reviewers. Any product that may be evaluated in this article, or claim that may be made by its manufacturer, is not guaranteed or endorsed by the publisher.
