# Supplementary material for: Plasticity of the Immune System in Children Following Treatment Interruption in HIV-1 Infection
Source: Front Immunol. 2021 Jul 29;12:643189. doi: 10.3389/fimmu.2021.643189 (PMC8406805; doi:10.3389/fimmu.2021.643189)
Supplement: Supplementary file 1 [file DataSheet_1.pdf]

Supplementary

Table 1S. Example of data input for the mathematical model to estimate thymic output

	TRECs/ CD4+, CD45RA+, CD45RO- T cell	Total number of naive CD4+, CD45RA+, CD31+ T cell population*	Ki67 of total CD4+, CD45RA+, CD31+ T cells as a fraction	Weight (kg)	c*	Δ**
**CT**						
**Week 0**	0,0145	1,478*10^11	0,0188	55	0,6	0,52
**Week 48**	0,0185	1,510*10^11	0,019	61	0,6	0,52
**Week 150**	0,0166	1,475*10^11	0,0131	77	0,6	0,52
**PTI**						
**Week 0**	0,0298	1,345*10^11	0,0078	40	0,6	0,52
**Week 12**	0,0574	8,228*10^10	0,0222	43	0,6	0,52
**Week 48**	0,0264	8,444*10^10	0,0188	47	0,6	0,52
**Week 150**	0,0303	2,045*10^11	0,0086	70	0,6	0,52

* Estimated as previously described, referring to the linear relationship between blood volume and body weight (15,16)

**Parameter values c and Δ were obtained as described previously (6).

Fig. 1S

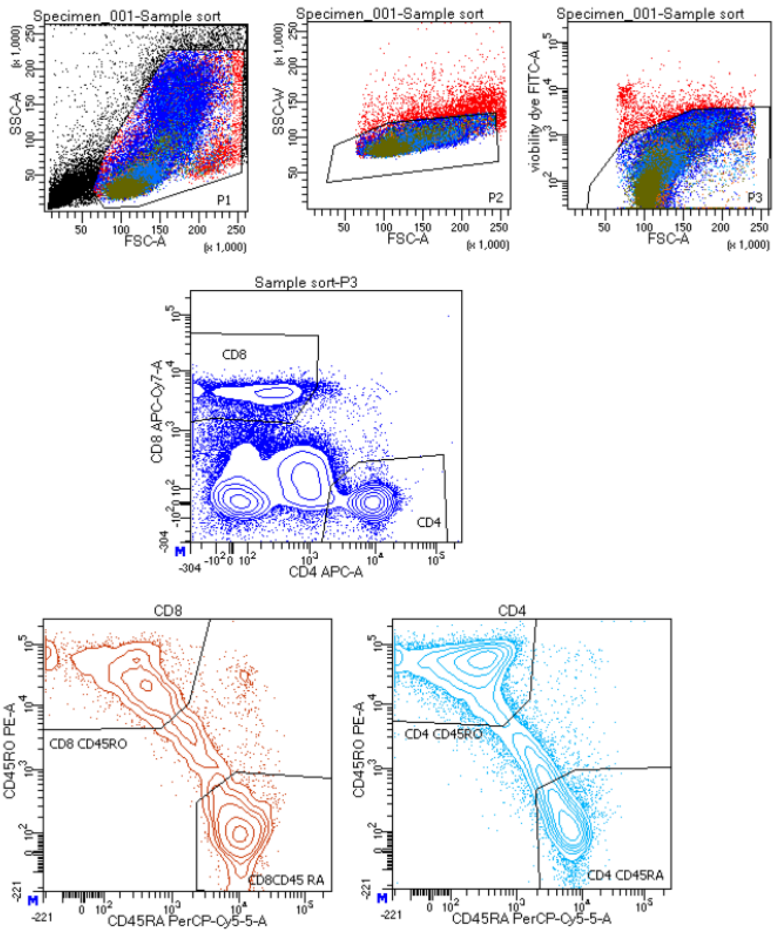

Fig. 1S Gating strategy for FACS sorting. Thawed PBMCs were FACS sorted to isolate and separate naïve $CD4^+$, $CD45RA^+$, $CD45RO^-$ T cells and memory $CD8^+$, $CD45RO^+$, $CD45RA^-$ T cell subsets for downstream high throughput sequencing with T cell specific primers.

Fig. 2S

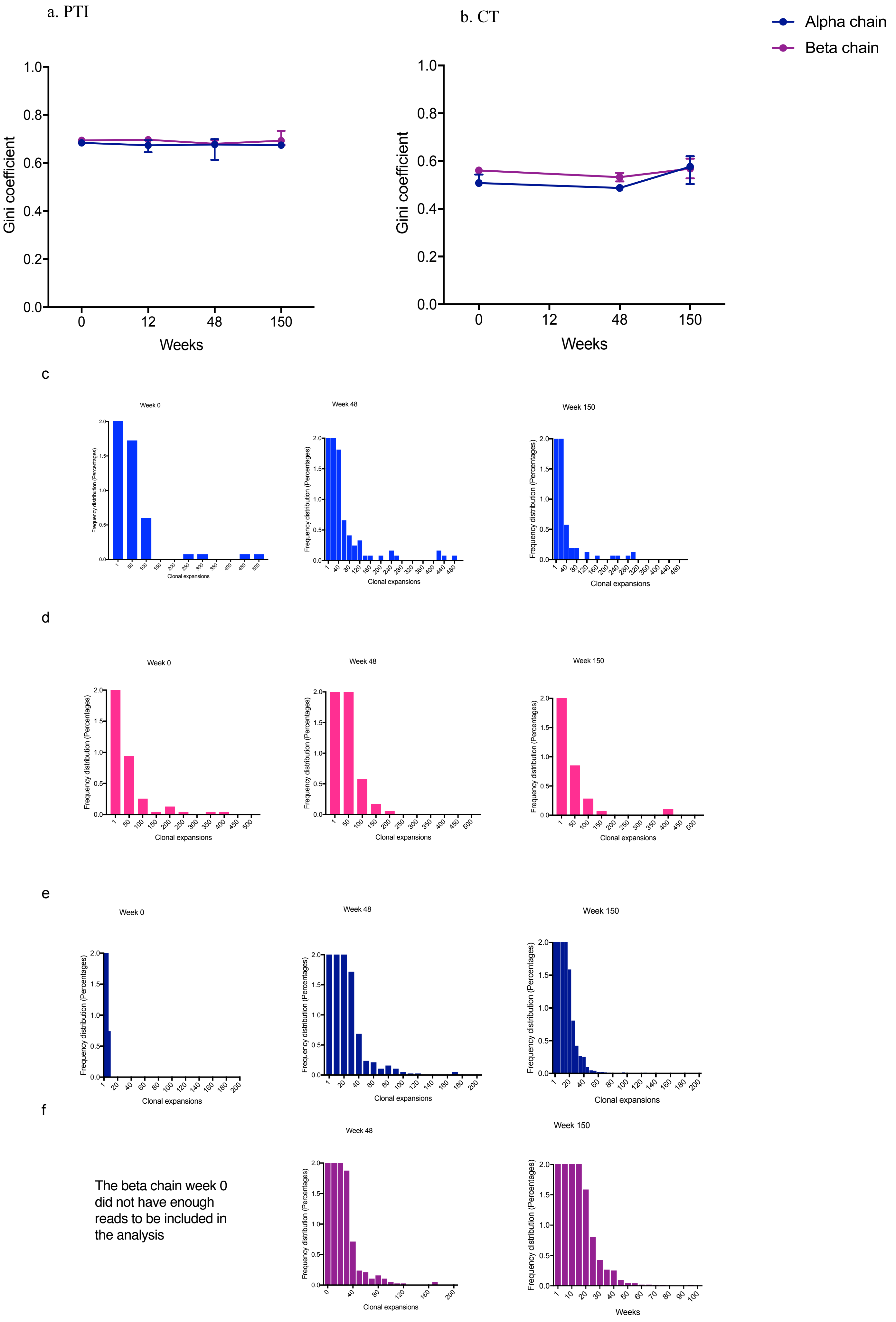

Figure 2S. Changes in TCR abundance distribution in memory CD8$^+$ T cells during ART treatment interruption. ***a-b***| *The degree of clonal expansion in the TCR repertoire measured using the Gini coefficient in TCR alpha (blue) and TCR beta sequences (purple) in the PTI (a) and CT (b) group.* ***c-f***| *TCR sequence abundance distribution in representative individuals.* ***c*** *and* ***e*** *TCR alpha;* ***d*** *and* ***f*** *TCR beta.* ***c*** *and* ***d*** *PTI group;* ***e*** *and* ***f*** *CT group.*

Fig. 3S

a

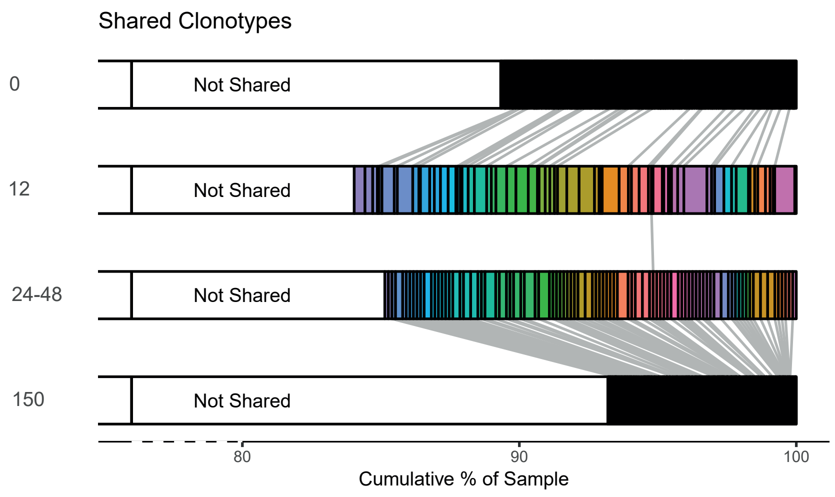

b

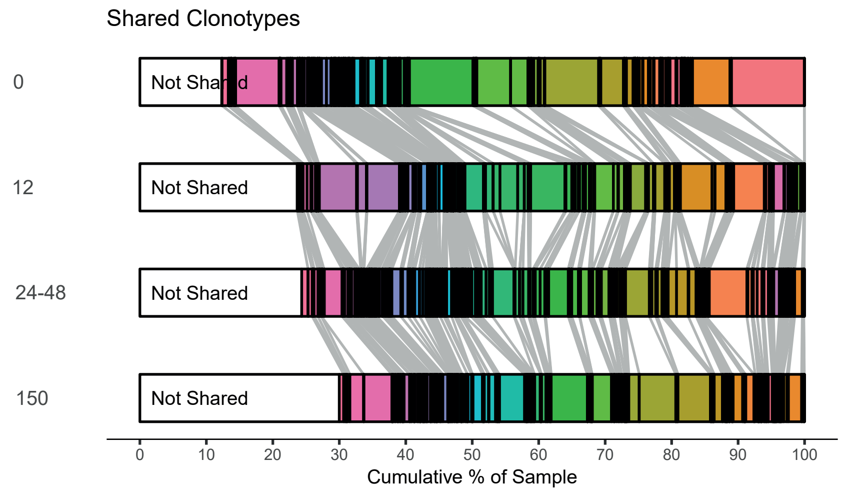

Fig. 3S. Repertoire dynamics and sequence sharing associated with ART interruption.
a| Shared CDR3s between weeks 0, 12, 24-48, and 150 originated from the naïve CD4+ T cell population shown as connecting grey lines. b| Shared CDR3s originated from the memory CD8+. The white horizontal bars represent the cumulative percentages of unique CDR3s and constitute the majority of the naïve TCR population. All CDR3s have been colour-coded alphabetically and the narrow coloured lines (black) represent very low frequency CDR3s. The same colour represents the same CDR3. No connecting grey lines between similar colours means that the sequences are conserved within the individual at least two time points, but are not shared between that pair of time points.

Table 2S

Table Analyzed	Paired t test data Nodes / total TCR clustered				
Two-way ANOVA	Ordinary				
Alpha	0.05				
Source of Variation	% of total variation	P value	P value summary	Significant?	
Patients	61.78	0.1615	ns	No	
Time	20.95	0.1525	ns	No	
ANOVA table	SS	DF	MS	F (DFn, DFd)	P value
Patients	0.0006083	3	0.0002028	F (3, 3) = 3.577	P=0.1615
Time	0.0002063	1	0.0002063	F (1, 3) = 3.638	P=0.1525
Residual	0.0001701	3	5.669e-005		
Number of missing values	0				
